# Supplementary material for: Prescription Patterns of Mycophenolate Mofetil in a Group of Patients from Colombia
Source: Healthcare (Basel). 2024 Nov 7;12(22):2224. doi: 10.3390/healthcare12222224 (PMC11593479; doi:10.3390/healthcare12222224)
Supplement: Supplementary file 1 [file healthcare-12-02224-s001.zip › healthcare-3161455-supplementary.pdf]

Supplementary Table S1. Patterns of use of mycophenolate mofetil in a group of patients affiliated with the Colombian Health System, according to the main indications identified, 2022

Medication	n	%	Mean dose (mg/day)	DDD[a]	Range (minimum and maximum dose) mg/day	Age (mean; SD)	Female Proportion (%)
**Systemic lupus erythematosus**							
Mycophenolate mofetil	383		1753 ± 726	0.87	500 - 4500	38.4 ± 13.7	91.4
500mg tablet	379	99.0	1764 ± 722	0.88	500 - 4500	38.5 ± 13.7	91.3
250mg tablet	4	1.0	750 ± 289	0.38	500 - 1000	36.8 ± 17.3	100.0
Main combinations							
Conventional DMARD + glucocorticoid	200	52.2	1800 ± 750	0.90	500 - 4500	36.9 ± 12.6	90.0
Conventional DMARD + Immunosuppressant + glucocorticoid	90	23.5	1711 ± 741	0.86	500 - 4000	38.3 ± 14.1	96.7
Glucocorticoid	44	11.5	1761 ± 576	0.88	500 - 3000	45.6 ± 16.0	90.9
Immunosuppressant + glucocorticoid	10	2.6	1300 ± 587	0.65	500 - 2000	40.6 ± 16.4	90.0
Other combinations	27	7.0	1667 ± 747	0.83	500 - 3000	36.2 ± 11.7	88.9
Mycophenolate mofetil monotherapy	12	3.1	1833 ± 718	0.92	500 - 3000	42.8 ± 14.6	83.3
**Solid organ transplant**							
Mycophenolate mofetil	63		1416 ± 594	0.71	500 - 3000	53.1 ± 16.6	76.2
500mg tablet	46	73.0	1533 ± 572	0.77	500 - 3000	52.6 ± 16.0	73.9
250mg tablet	17	27.0	1103 ± 552	0.55	500 - 2000	54.7 ± 18.5	82.4
Main combinations							
Conventional DMARD + glucocorticoid	0	0.0	-	-	-	-	-
Conventional DMARD + Immunosuppressant + glucocorticoid	2	3.2	1250 ± 354	0.63	1000 - 1500	57.0 ± 7.1	100.0
Glucocorticoid	4	6.3	875 ± 250	0.44	500 - 1000	52.2 ± 15.0	100.0
Immunosuppressant + glucocorticoid	29	46.0	1483 ± 619	0.74	500 - 2500	52.1 ± 16.2	79.3
Other combinations	22	34.9	1420 ± 614	0.71	500 - 3000	51.8 ± 18.5	63.6
Mycophenolate mofetil monotherapy	6	9.5	1500 ± 548	0.75	1000 - 2000	62.7 ± 15.0	83.3
**Other rheumatic diseases**							
Mycophenolate mofetil	83		1464 ± 604	0.73	500 - 4000	56.8 ± 15.8	92.8
500mg tablet	83	100.0	1463 ± 604	0.73	500 - 4000	56.8 ± 15.8	92.8
250mg tablet	0	0.0	-	-	-	-	-
Main combinations							
Conventional DMARD + glucocorticoid	20	24.1	1475 ± 545	0.74	500 - 2000	48.7 ± 15.9	90.0
Conventional DMARD + Immunosuppressant + glucocorticoid	5	6.0	1300 ± 447	0.65	1000 - 2000	55.6 ± 23.3	100.0
Glucocorticoid	16	19.3	1562 ± 929	0.78	500 - 4000	57.2 ± 15.8	93.8
Immunosuppressant + glucocorticoid	11	13.3	1545 ± 610	0.77	1000 - 3000	56.6 ± 17.0	90.9
Other combinations	12	14.5	1375 ± 483	0.69	500 - 2000	61.1 ± 12.9	100.0
Mycophenolate mofetil monotherapy	19	22.9	1421 ± 449	0.71	500 - 2500	62.8 ± 12.5	89.5

**Nephrotic syndrome**							
Mycophenolate mofetil	73		1616 ± 710	0.81	500 - 4000	45.4 ± 16.4	84.9
500mg tablet	72	98.6	1632 ± 702	0.82	500 - 4000	45.2 ± 16.4	84.7
250mg tablet	1	1.4	500	0.25	500	59.0	100.0
Main combinations							
Conventional DMARD + glucocorticoid	16	21.9	1938 ± 574	0.97	1000 - 3000	39.2 ± 11.7	87.5
Conventional DMARD + Immunosuppressant + glucocorticoid	6	8.2	1500 ± 547	0.75	1000 - 2000	36.8 ± 20.5	83.3
Glucocorticoid	16	21.9	1781 ± 930	0.89	500 - 4000	49.4 ± 15.5	87.5
Immunosuppressant + glucocorticoid	6	8.2	1333 ± 516	0.67	1000 - 2000	31.7 ± 22.4	83.3
Other combinations	13	17.8	1461 ± 776	0.73	500 - 3000	50.7 ± 12.5	76.9
Mycophenolate mofetil monotherapy	16	21.9	1406 ± 554	0.70	1000 - 2500	51.8 ± 15.9	87.5

DMARD: Disease-Modifying Antirheumatic Drug; DDD: Defined daily dose; SD: Standard deviation; IQR: Interquartile range. [a]Proportion between the mean daily dose received and the defined daily dose.
